# Postoperative Surgical Site and Secondary Infections in Colorectal Cancer Patients With a History of SARS-CoV-2: A Retrospective Cohort Study

**DOI:** 10.7759/cureus.78077

**Published:** 2025-01-27

**Authors:** Dhierin R Jagdewsing, Noor Safra C Fahmy, Xin Chen, Yanick K Keuzetien, FHNS Anthony Silva, Haonan Kang, Yang Xu, Abdulkarem Al-sharabi, Shruti A Jagdewsing, Sima A Jagdewsing

**Affiliations:** 1 Department of Hernia and Colorectal Cancer, Second Affiliated Hospital of Dalian Medical University, Dalian, CHN; 2 Department of Clinical Medicine, Dalian Medical University, Dalian, CHN; 3 Department of Spine Surgery, Southern Medical University, Guangzhou, CHN; 4 Department ofClinical Medicine, Dalian Medical University, Dalian, CHN; 5 General Practice, Anton de Kom University of Suriname, Paramaribo, SUR; 6 Department of Neurology, Curaçao Medical Center, Willemstad, CUW

**Keywords:** colorectal cancer, covid-19, pandemic, secondary infections, ssi, surgical site infection

## Abstract

Background: The outbreak of the COVID-19 pandemic brought unique challenges to the field of healthcare, particularly to the surgical field. This retrospective cohort study aims to compare the risk of surgical site infection (SSI) and secondary infection between colorectal cancer (CRC) patients with a history of COVID-19 infection and those without.

Method: A cohort of 200 CRC patients, comprising 100 with a documented history of COVID-19 infection and 100 without, were retrospectively analyzed. Independent sample t-tests for continuous variables, Chi-square tests for categorical variables, and additionally univariate and multivariate binary logistic regression analysis were performed using IBM SPSS Statistics for Windows, Version 29.0.2 (Released 2023; IBM Corp., Armonk, New York, United States). The data were collected from the medical records of patients treated at the Hernia and Colorectal Surgery department of the Second Affiliated Hospital of Dalian Medical University, Dalian, China. Key clinical variables examined included the incidence of SSIs, occurrence of secondary infections, presence of comorbidities such as diabetes and hypertension, and duration of hospitalization.

Results: The comparative analysis yielded compelling differences between CRC patients with a history of COVID-19 infection and those without. The study revealed a significantly higher incidence of SSI (68.8% vs. 31.3%, p=0.003) and secondary infection (70.1% vs. 29.9%, p<0.001) among patients with a history of COVID-19. In the multivariate analysis for SSIs, hypertension (OR = 2.78, 95%CI: 1.03-7.54, p=0.044) and surgical procedure type (open vs. laparoscopic) (OR = 6.04, 95%CI: 1.88-19.43, p=0.003) were found to be significant independent predictors. Patients with a history of COVID-19 had a significantly higher incidence of secondary infections (70.1% vs. 29.9%, p<0.001), with multivariate analysis showing COVID-19 status (OR: 3.053, 95%CI: 1.515-6.154, p=0.002), hypertension (OR: 2.632, 95% CI: 1.154-6.006, p=0.021), and diabetes mellitus (OR: 4.326, 95%CI: 2.029-9.226, p<0.001) as independent risk factors.

Conclusion: This study highlights significant insight into SSI rates, secondary infection rates, and clinical characteristics between CRC patients with and without a history of COVID-19 infection. The findings underscore that CRC patients with hypertension who underwent open surgery procedures exhibited a higher susceptibility to SSI. Following CRC patients in combination with comorbidities such as hypertension, diabetes, and a history of COVID-19 infection exhibited higher susceptibility to secondary infections. This study contributes to the evolving understanding of the impact of COVID-19 history on surgical outcomes, providing valuable insight to healthcare providers in optimizing care for CRC patients in the context of this ongoing health crisis.

## Introduction

Colorectal cancer (CRC), a malignant tumor originating in the colon or rectum, is one of the most significant global health challenges, ranking as the third most diagnosed cancer and the second leading cause of cancer-related deaths worldwide [1]. Its development often stems from genetic and environmental factors, including a family history of CRC, genetic syndromes like familial adenomatous polyposis (FAP) and Lynch syndrome, aging, and lifestyle factors such as a high-fat diet, smoking, and sedentary habits [2]. CRC typically progresses through a series of genetic mutations and epigenetic changes, beginning with benign adenomatous polyps that may mutate into invasive adenocarcinomas through alterations in oncogenes and tumor suppressor genes such as *APC*, *KRAS*, and *TP53* [2]. Early symptoms are often non-specific and include changes in bowel habits, rectal bleeding, and abdominal discomfort, with advanced stages presenting more severe symptoms like bowel obstruction or metastasis to organs such as the liver and lungs [3]. Diagnosis relies on colonoscopies, sigmoidoscopies, and advanced imaging modalities, while treatments range from surgical resection to systemic therapies like chemotherapy, targeted agents (e.g., anti-vascular endothelial growth factor (VEGF) antibodies), and immunotherapy [4]. Advances in laparoscopic and robotic surgeries have reduced recovery times and postoperative complications [5]. Despite improvements, CRC remains a critical public health issue with regional disparities, higher incidence in developed countries, and increasing rates among younger populations [6,7]. Prognosis is largely stage-dependent, with early detection offering high cure rates and advanced stages requiring aggressive treatment [8]. Global trends indicate a rising burden due to aging populations and lifestyle transitions, necessitating comprehensive strategies focused on prevention, early detection, and equitable access to care [9,10].

The COVID-19 pandemic has significantly disrupted CRC management, exacerbating patient vulnerabilities. A systematic review by Mazidimoradi et al. highlighted that decreased healthcare activity, resource reallocation, and patient reluctance to seek medical help led to significant risks for CRC patients [11]. Additionally, studies by Clifford et al. [12] and Ren et al. [13] discussed the challenges in CRC management during the COVID-19 epidemic, emphasizing that the pandemic necessitated rapid adaptations in CRC services, including restructuring clinical teams and modifying treatment protocols, to maintain patient care amidst unprecedented challenges; these studies highlight the need for resilient healthcare strategies to ensure continuity of care for vulnerable populations. Many CRC patients experienced delays in treatments like chemotherapy or radiation therapy, which were tailored to minimize infection risks. Telemedicine emerged as a valuable alternative for consultations and follow-ups, though disparities in digital access and literacy posed barriers, especially in rural or underprivileged areas [14,15].

Surgical site infections (SSIs), already a significant complication in CRC surgeries, were compounded by pandemic-induced resource constraints. SSIs, caused by pathogens at the surgical site, vary in severity and can lead to prolonged hospital stays, delayed adjuvant therapy, or life-threatening complications like sepsis [16]. Risk factors include patient-related elements like age, obesity, diabetes, and immunosuppression, alongside surgical and postoperative factors such as prolonged operative times and inadequate wound care [17]. SSIs not only compromise immediate recovery but also impact long-term outcomes, increasing recurrence risks and diminishing survival rates [18]. Beyond physical health, SSIs and COVID-19-related disruptions significantly affect mental well-being, with patients experiencing heightened anxiety, depression, and isolation. Comprehensive care, including patient education on wound care and lifestyle adjustments, alongside robust infection control measures, is critical in mitigating these impacts [10].

Postoperative secondary infections, occurring at sites other than the surgical area, are significant complications for CRC patients. These infections include urinary tract infections (UTIs), pneumonia, bloodstream infections, and *Clostridioides difficile* infections. Factors contributing to these complications encompass fecal contamination during surgery, prolonged use of invasive devices, and the patient's overall health status. Implementing effective perioperative care such as appropriate antibiotic prophylaxis and stringent infection control measures is crucial to mitigate these risks [19]. The interplay of CRC’s clinical challenges and pandemic disruptions underscores the urgent need for resilient healthcare systems capable of adapting to crises while ensuring equitable, high-quality cancer care [5,8]. Also, previous studies emphasize the importance of addressing comorbidities in CRC patients, as conditions like hypertension and diabetes can further impair immune function, comprise wound healing, and increase the risk of infections [20]. This retrospective cohort study aims to investigate and compare the risk of SSI rates and secondary infections between CRC patients with a history of COVID-19 infection and those without.

## Materials and methods

This was a retrospective cohort study conducted to assess the outcomes of SSIs and secondary infections of CRC surgeries in patients with and without a history of COVID-19. The study utilized medical records from the Inguinal Hernia and Colorectal Cancer Department at the Second Affiliated Hospital of Dalian Medical University, Dalian, China, covering cases between 2019 and 2024. This robust study design enabled the comprehensive evaluation of the interplay between CRC and prior COVID-19 infection, offering insights into the unique challenges and considerations for surgical outcomes in this patient population.

Patient selection

The study included adult patients (≥18 years) diagnosed with colon or rectal cancer who underwent surgery between 2019 and 2024, with complete medical records detailing comorbidities and perioperative outcomes. Patients were grouped based on a history of COVID-19 infection. Exclusions included incomplete records, non-surgical treatments, unrelated recent surgeries, active SARS-CoV-2 infection during surgery, and other cancers. A total of 200 patients were analyzed, with equal representation of 100 patients in two groups, one with a documented history of COVID-19 (Group 1) and one without (Group 2).

Data collection

Key demographic variables such as age, gender, and body mass index (BMI) were recorded alongside comorbidities, including hypertension, diabetes, heart conditions such as coronary artery disease, heart failure, or arrhythmias, and degenerative lung diseases such as chronic obstructive pulmonary disease (COPD), pulmonary fibrosis, or emphysema. Additionally, prior surgical histories, specific CRC diagnoses, and categorization as colon or rectal cancer were included. The surgical techniques (open or laparoscopic) were documented to evaluate their association with surgical outcomes. Rigorous data extraction ensured that all relevant preoperative and postoperative variables were captured, providing a solid foundation for the comparative analysis of the two cohorts.

Postoperative outcomes, a crucial aspect of this study, included the incidence of SSIs, secondary infections (pneumonia, UTIs, and catheter-associated infections), volume of intra-operative blood loss, surgery duration, and severity of postoperative pain based on a numeric rating scale (NRS) where 1-3 is mild pain, 4-6 moderate pain, 7-10 severe pain. Other metrics, such as hospitalization duration and recovery timelines, were also analyzed to understand the broader impact of COVID-19 history on surgical recovery.

Data analysis

The data was analyzed using IBM SPSS Statistics for Windows, Version 29.0.2 (Released 2023; IBM Corp., Armonk, New York, United States). Independent Sample t-tests were performed for continuous variables and Chi-square tests for categorical variables. The tests generated p-values to assess significant differences between the group with COVID-19 history and the group without COVID-19 history, with significance set at p < 0.05. Additionally, univariate binary logistic regression analysis was conducted to evaluate the association between individual predictors and the risk of SSIs and secondary infections. Variables that were significant in the univariate analysis (p < 0.05) were included in a multivariate binary logistic regression model to identify independent predictors while adjusting for potential confounders. Odds ratios (ORs) with 95% confidence intervals (CIs) and p-values were reported to assess the strength and significance of these associations.

## Results

Table 1 shows the comparative analysis of CRC patients with a history of COVID-19 (Group 1) and without a history of COVID-19 (Group 2), which reveals several significant differences across demographic, clinical, and surgical parameters. Gender distribution showed no statistical significance, with male and female patients nearly equally represented in both groups (p = 0.394). Age also demonstrated no significant variation between the two groups (p = 0.096). However, significant differences were observed in weight (p < 0.001) and height (p = 0.026), where patients with COVID-19 history had higher mean values. BMI also showed a slight but statistically significant increase in patients without COVID-19 history (p = 0.003). Comorbidities such as hypertension, diabetes, and heart conditions were notably more prevalent in the group with COVID-19 history (p = 0.011, p < 0.001, and p < 0.001, respectively). Although lung conditions were more common in the group with COVID-19 history, the difference did not reach statistical significance (p = 0.071). A history of previous surgeries was significantly higher in Group 1 (p = 0.050). No significant difference was observed in the distribution of colon and rectal cancer between the two groups (p = 0.558). However, the type of surgical procedure varied significantly, with Group 1 more likely to undergo open surgery compared to laparoscopic procedures, which were more common in Group 2 (p = 0.013).

**Table 1:** Comparison between the CRC patients with COVID-19 history and without COVID-19 history (N=200) CRC: colorectal cancer

Variables	CRC patients with COVID-19 history (n=100)	CRC patients without COVID-19 history (n=100)		P-value
Gender, n (%)				0.394
Male	52 (47.3)		58 (52.7)	
Female	48 (53.3)		42 (46.7)	
Age, mean ± SD	65.88 ± 10.50		63.95 ± 10.34	0.096
Weight (kg), mean ± SD	67.8 ± 4.84		70.1 ± 4.40	<0.001
Height, mean ± SD	1.67 ± 0.52		1.69 ± 0.043	0.026
BMI, mean ± SD	24.35 ± 1.23		24.78 ± 0.99	0.003
Hypertension, n (%)	74 (56.5)		57 (43.5)	0.011
Diabetes, n (%)	58 (65.2)		31 (34.8)	<0.001
Heart conditions, n (%)	35 (76.1)		11 (23.9)	<0.001
Lung conditions, n (%)	15 (68.2)		7 (31.8)	0.071
Previous surgery, n (%)	31 (62.0)		19 (38.0)	0.050
Colorectal Cancer, n (%)				0.558
Colon cancer	65 (51.6)		61 (48.4)	
Rectal cancer	35 (47.3)		39 (52.7)	
Surgery procedure, n (%)				0.013
Open	14 (77.8)		4 (22.2)	
Laparoscopic	86 (44.3)		96 (52.7)	

Table 2 shows the comparison of postoperative outcomes between the two groups, revealing significant differences in certain parameters. SSIs were significantly more common in Group 1 (68.8%) compared to Group 1 (31.3%, p = 0.003). Similarly, secondary infections were markedly higher in Group 1 (70.1%) than in Group 2 (29.9%, p < 0.001). While the severity of post-surgical pain varied, the difference between groups did not reach statistical significance (p = 0.094). Hospitalization duration was significantly longer for Group 1, with a mean of 18.89 days compared to 15.50 days for Group 2 (p = 0.016). However, there were no significant differences in blood loss (p = 0.413) or surgery duration (p = 0.143) between the groups. These findings highlight the elevated risk of postoperative infections and extended recovery times in CRC patients with a history of COVID-19, emphasizing the need for vigilant perioperative care and infection prevention strategies in this population.

**Table 2:** Comparison of postoperative outcomes between CRC patients with COVID-19 history and without COVID-19 history (N=200) SSI: surgical site infection; CRC: colorectal cancer

Variables	CRC patients with COVID-19 history (n=100)	CRC patients without COVID-19 history (n=100)	P-value
SSI, n (%)	33 (68.8)	15 (31.3)	0.003
Secondary Infections, n (%)	61 (70.1)	26 (29.9)	<0.001
Post-surgical pain, n (%)			0.094
Mild	31 (50.8)	30 (49.2)	
Moderate	37 (42.5)	50 (57.5)	
Severe	32 (61.5)	20 (38.5)	
Hospitalization (days), mean ± SD	18.89 ± 12.85	15.50 ± 9.12	0.016
Blood loss (ml), mean ± SD	208.59 ± 80.93	206.10 ± 78.21	0.413
Surgery duration (minutes), mean ± SD	212.32 ± 54.06	204.73 ± 45.86	0.143

**Figure 1:**
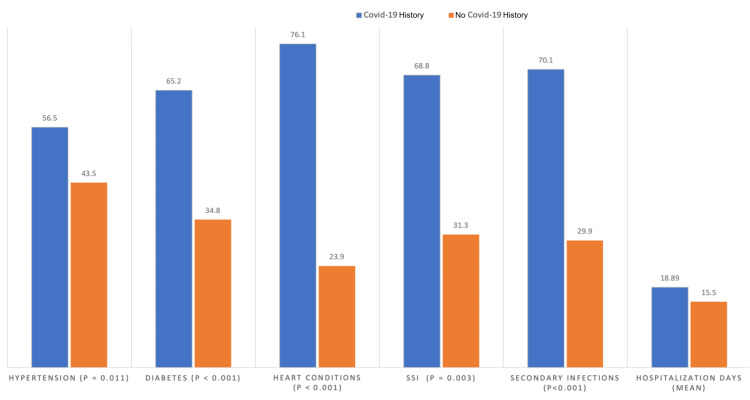
Comparison of the impact of COVID-19 on key surgical metrics and outcomes between the two groups (N=200) Values given in percentages

Figure 1 shows a clustered bar chart that highlights statistically significant differences between the two groups across key metrics. Hypertension (56.5% vs. 43.5%) (p=0.011), diabetes (65.2% vs. 34.8%) (p < 0.001), and heart conditions (76.1% vs. 23.9%) (p < 0.001), were notably more prevalent in Group 1, indicating a strong association between these comorbidities and COVID-19 infection. SSIs (68.8% vs. 31.3%) (p=0.003) and secondary infections (70.1% vs. 29.9%) (p<0.001) were also significantly higher in Group 1, reflecting the challenges of managing postoperative complications in these patients. Additionally, hospitalization duration was longer in Group 1 (18.9 days) compared to Group 2 (15.5 days) (p=0.016), highlighting the extended care required for this group.

Table 3 shows the results of univariate and multivariate logistic regression analysis for risk factors associated with SSI in CRC patients. ORs greater than 1 indicate an increased risk of SSIs. The univariate model shows the unadjusted ORs, 95% CIs, and p-values for each variable, with open surgery showing the highest risk (OR = 8.11, 95% CI: 2.85-23.08, p < 0.001) and COVID-19 history status also significant (OR = 2.79, 95%CI: 1.40-5.56, p = 0.004). In the multivariate model, adjusted for all predictors, hypertension (OR = 2.78, 95%CI: 1.03-7.54, p = 0.044) and surgical procedure (OR = 6.04, 95%CI: 1.88-19.43, p = 0.003) remain significant, while COVID-19 history status (OR = 1.57, 95%CI: 0.72-3.39, p = 0.254) loses significance.

**Table 3:** Univariate and multivariate binary logistic regression models for the risk of SSI (N=200) SSI: surgical site infection

Variables	OR	95% CI	P-value
Univariate Model			
COVID-19 history vs no COVID-19 history	2.79	1.40-5.56	0.004
Hypertension	4.04	1.70-9.58	0.002
Diabetes mellitus	4.28	2.12-8.67	<0.001
Heart conditions	3.95	1.94-8.07	<0.001
Lung Conditions	2.47	0.99-6.20	0.055
Open vs laparoscopic surgery	8.11	2.85-23.08	<0.001
Multivariate Model			
COVID-19 history vs no COVID-19 history	1.57	0.72–3.39	0.254
Hypertension	2.78	1.03–7.54	0.044
Diabetes mellitus	1.67	0.72–3.89	0.232
Heart conditions	2.04	0.86–4.83	0.106
Lung conditions	1.05	0.37–2.95	0.934
Open vs. laparoscopic surgery	6.04	1.88–19.43	0.003

Table 4 shows the univariate and multivariate binary logistic regression models for the risk of secondary infections. In the univariate analysis, significant associations were observed for COVID-19 status (OR: 4.452, 95%CI: 2.441-8.119, p<0.001), hypertension (OR: 4.945, 95%CI: 2.504-9.765, p<0.001), diabetes mellitus (OR: 8.784, 95%CI: 4.620-16.701, p<0.001), heart conditions (OR: 4.701, 95%CI: 2.283-9.678, p<0.001), lung conditions (OR: 5.246, 95%CI: 1.851-14.863, p=0.002), and open versus laparoscopic surgery procedure (OR: 7.639, 95%CI: 2.135-27.329, p=0.002). In the multivariate analysis, significant associations remained for COVID-19 status (OR: 3.053, 95%CI: 1.515-6.154, p=0.002), hypertension (OR: 2.632, 95% CI: 1.154-6.006, p=0.021), and diabetes mellitus (OR: 4.326, 95%CI: 2.029-9.226, p<0.001). Heart conditions, lung conditions, and type of surgery were not significantly associated with secondary infection risk in the multivariate model.

**Table 4:** Univariate and multivariate binary logistic regression models for the risk of secondary infections (N=200)

Variables	OR	95% CI	P-value
Univariate Model			
COVID-19 history vs no COVID-19 history	4.452	2.441 – 8.119	<0.001
Hypertension	4.945	2.504 – 9.765	<0.001
Diabetes mellitus	8.784	4.620 – 16.701	<0.001
Heart conditions	4.701	2.283 – 9.678	<0.001
Lung conditions	5.246	1.851 – 14.863	0.002
Open vs. laparoscopic surgery procedure	7.639	2.135 – 27.329	0.002
Multivariate Model			
COVID-19 history vs no COVID-19 history	3.053	1.515 – 6.154	0.002
Hypertension	2.632	1.154 – 6.006	0.021
Diabetes mellitus	4.326	2.029 – 9.226	<0.001
Heart conditions	1.157	0.471 – 2.841	0.750
Lung conditions	1.996	0.609 – 6.543	0.254
Open vs. laparoscopic surgery	3.410	0.790 – 14.712	0.100

## Discussion

This study compared various demographic and clinical factors of CRC patients with and without COVID-19 history. Our findings indicated several significant differences between the two groups. There was a higher prevalence of comorbidities among patients with COVID-19 history. Hypertension affected 74 patients (56.5%) in the COVID-19 history group, compared to 57 patients (43.5%) (p=0.011) in the group without COVID-19 history. Similarly, diabetes was more common in patients with COVID-19 history, with 58 (65.2%) compared to 31 (34.8%) (p<0.001) in the group without COVID-19 history. Heart conditions were also significantly more prevalent among patients with COVID-19 history, with 35 (76.1%) affected compared to 11 (23.9%) (p<0.001) in the group without COVID-19 history.

SSIs were another notable finding. In the group with COVID-19 history, 33 patients (68.8%) experienced SSIs, contrasting sharply with 15 patients (31.3%) (p=0.003) in the group without COVID-19. Univariate and multivariate logistic regression analyses were performed to further investigate the factors contributing to SSIs. Univariate analysis for the risk of SSI identified significant associations for COVID-19 status, hypertension, diabetes mellitus, heart conditions, and surgical procedure type (open vs. laparoscopic), with open surgery posing the highest risk (OR: 8.11, 95%CI: 2.85-23.08). In the multivariate model, hypertension (OR: 2.78, 95%CI: 1.03-7.54, p = 0.044) and open surgical procedure (OR: 6.04, 95%CI: 1.88-19.43, p = 0.003) were the only significant independent predictors, while COVID-19 status, diabetes, and other comorbidities lost significance, likely due to confounding or shared variance. These results suggest that CRC patients with hypertension who underwent open surgery had a higher risk of developing SSIs. This finding is in line with a study by Zhang et al., which found that patients with a prior diagnosis of hypertension had a significantly increased risk of developing SSIs after open colorectal surgery procedures [21]. This emphasizes the importance of managing hypertension in the perioperative period to mitigate this risk.

Further, the univariate analysis identified significant associations between secondary infections and factors such as COVID-19 history status, hypertension, and diabetes mellitus. However, in our multivariate analysis, only the group with COVID-19 history (OR: 3.053; 95%CI: 1.515-6.154; P = 0.002), hypertension (OR: 2.632; 95%CI: 1.154-6.006; P = 0.021), and diabetes mellitus (OR: 4.326; 95%CI: 2.029-9.226; P < 0.001) remained significant predictors of postoperative secondary infections. This suggests that patients with comorbidities such as hypertension and diabetes with a history of COVID-19 and who underwent CRC surgery are at higher risk of developing secondary infections. These findings are consistent with existing literature that highlights the increased risk of postoperative complications, including secondary infections, among patients with recent COVID-19 infections, likely due to the virus's impact on immune and inflammatory responses [22].

Hospitalization duration also varied significantly, with patients having COVID-19 history having a longer average stay of 18.89 days ± 12.85 compared to 15.50 days ± 9.12 (p=0.016) for patients without COVID-19 history. This is due to the challenges faced by the patients who have had COVID-19, as they often experience prolonged recovery times. A study mentioned that COVID-19 can cause lasting respiratory and immune system impairment that delays recovery and complicates postoperative care, leading to longer hospital stays and increased healthcare resource utilization [23]. Further, the type of surgery also plays a significant role in the risk of SSIs. Studies have shown that open surgeries are more likely to result in increased SSIs due to larger incisions, increased tissue trauma, and greater pathogen exposure during surgery. On the other hand, minimally invasive techniques such as laparoscopic and robotic-assisted surgeries are associated with lower rates of SSIs due to reduced tissue disruption and shorter recovery times [24,25]. So, due to the pandemic, patients may benefit from these less invasive procedures to reduce the risk of infection, particularly since they are already at a heightened risk due to their history of viral infection and underlying comorbidities.

The increased susceptibility to SSIs and secondary infections in CRC patients with hypertension and diabetes who underwent open surgery underscores the need for targeted infection prevention strategies and perioperative care. As highlighted by some researchers, these patients require tailored perioperative care, including precise preoperative screening for COVID-19, judicious antimicrobial prophylaxis, and individualized surgical planning [22,23,26]. The combination of COVID-19-related immune suppression, comorbidities, and the complex nature of CRC treatments necessitates a comprehensive, personalized approach to care. By implementing stringent infection control measures, utilizing minimally invasive surgical techniques, and offering tailored perioperative care, healthcare providers can improve outcomes and reduce complications in this population. Further research, particularly long-term studies and randomized controlled trials is needed to better understand the lasting effects of COVID-19 on CRC surgery outcomes and to refine clinical protocols. Enhanced protocols, such as preoperative COVID-19 testing, shorter hospital stays, and minimally invasive techniques, as discussed by Di Saverio et al. [27], will be critical in optimizing care and improving patient outcomes moving forward.

Limitations and recommendations

Despite the valuable insights provided by this study, several limitations should be acknowledged. First, the retrospective nature of the study may introduce selection bias and limit the ability to establish causality between COVID-19 and increased SSI risk. Second, the sample size, while sufficient to identify significant differences, may not capture the full variability of outcomes among CRC patients with a history of COVID-19. Third, the heterogeneity in surgical techniques, postoperative care, and patient demographics across study settings may affect the generalizability of these findings. Furthermore, the study did not account for the potential effects of COVID-19 variants, vaccination status, or the timing of infection relative to surgery, all of which could influence patient outcomes. Therefore, future prospective studies with larger, more diverse cohorts and standardized protocols are needed to validate these findings and address these limitations.

Several clinical implications emerge from this research, highlighting the necessity of a multidisciplinary approach involving surgeons, infectious disease specialists, and primary care providers in managing CRC patients with a history of COVID-19. Effective strategies to mitigate the risk of SSIs include comprehensive screening for comorbidities, vigilant perioperative care, and the use of appropriate safety equipment. Individualized infection prevention measures, based on scientific data, are critical for this vulnerable group, encompassing standardized protocols for preparing the operating area, appropriate antibiotic prescribing, and diligent postoperative surveillance. These strategies not only help prevent the emergence of SSIs but also alleviate pressure on healthcare resources, allowing for enhanced care for CRC patients with a history of COVID-19.

## Conclusions

This study demonstrates that CRC patients with hypertension who underwent open surgery are at significantly higher risk for SSIs. Further, CRC patients with comorbidities such as hypertension, diabetes, and a history of COVID-19 are at higher risk of developing secondary infections. These findings emphasize the importance of preoperative screening, addressing comorbidities, and prioritizing minimally invasive surgical techniques to mitigate SSI risk and improve postoperative outcomes. By tailoring perioperative care to individual patient needs, including those with a history of COVID-19, healthcare providers can reduce complications and enhance recovery. Future research should focus on validating these findings in larger cohorts and exploring long-term outcomes to refine clinical protocols.
